# Molecular Typing of Hospital-Acquired *Staphylococcus aureus* Isolated from Isfahan, Iran

**DOI:** 10.1155/2014/185272

**Published:** 2014-11-06

**Authors:** Seyed Asghar Havaei, Fahimeh Ghanbari, Ali Asghar Rastegari, Amir Azimian, Farzad Khademi, Nafiseh Hosseini, Amirmorteza Ebrahimzadeh Namvar, Hamid Vaez, Seyed Mehdi Havaei, Mojtaba Shahin

**Affiliations:** ^1^Department of Microbiology, School of Medicine, Isfahan University of Medical Sciences, Isfahan, Iran; ^2^Department of Microbiology, Faculty of Bioscience, Islamic Azad University, Falavarjan Branch, Isfahan, Iran; ^3^Department of Molecular and Cell Biochemistry, Faculty of Bioscience, Falavarjan Branch, Islamic Azad University, Isfahan, Iran; ^4^Department of Pathobiology and Anatomy, School of Medicine, North Khorasan University of Medical Science, Bojnord, Iran; ^5^Department of Medical Bacteriology and Virology, Ghaem Hospital, School of Medicine, Mashhad University of Medical Sciences, Mashhad, Iran; ^6^Department of Biology, Faculty of Sciences, Shiraz University, Shiraz, Iran; ^7^Department of Microbiology, School of Medicine, Ahvaz University of Medical Sciences, Ahvaz, Iran

## Abstract

*Background*. *Staphylococcus aureus* (*S. aureus*) is one of the most common pathogens that cause hospital- and community-acquired infections in the world. The use of molecular typing methods is essential for determining the origin of the strains, their clonal relations, and also in epidemiological investigations. The purpose of this study was to determine the prevalence of antibiotic resistant *S. aureus* isolates and using spa, agr, and SCCmec typing to determine the dominant types in Iran. *Material and Method*. Fifty isolates of *S. aureus* were collected from January to May 2010. *S. aureus* identification was performed by biochemical tests. Disk diffusion method was employed to assess the sensitivity of *S. aureus* strains to antibiotics and then genetic analysis of bacteria was performed using SCCmec, agr, and spa typing. *Results*. *S. aureus* resistance to tetracycline, cefoxitin, clindamycin, ciprofloxacin, gentamicin, Cot: cotrimoxazole, levofloxacin, rifampin, and vancomycin were found to be 36%, 18%, 12%, 12%, 22%, 6%, 6%, and 0%, respectively. The results of this study showed that 16% of the isolates were resistant to methicillin (MRSA) and the majority of isolates were SSC mec type IV. In addition spa and agr typing revealed agr typeI and spa type t7688 to be the most predominant. *Conclusion*. In this study, spa typing showed 100% reliability and the t7688 spa type had a frequency of 26% compared to the frequency of 0.0% in the Ridom SpaServer. The frequency of t304 spa type was higher than the global average.

## 1. Introduction

*Staphylococcus aureus* (*S. aureus*) is one of the most prevalent pathogens that cause both community and nosocomial acquired infections and can produce a wide variety of diseases from skin surface infections, such as folliculitis and furunculosis, to life threating conditions, such as endocarditis, pneumonia, and septicemia [1–3]. The expression of many virulence factors in* S. aureus *is under control of the agr system and to date four major agr types in* S. aureus *have been recognized [4, 5]. Resistance to methicillin is due to* mecA* gene that is part of the* staphylococcal* cassette chromosome. This gene encodes the protein PBP2A (protein binding to penicillin) that inhibits the action of *β*-lactam antibiotics. SCCmec elements have been classified into eight different types (I–VIII) and some of them are divided further into subtypes [6, 7]. The increasing antibiotic resistance in this bacterium is a major concern that underlines the importance of the use of efficient typing methods for monitoring and limiting the spread of epidemic clones between hospitals [1, 8, 9]. Of the various molecular methods, PFGE, due to its high differentiation potential, was considered the gold standard for strain typing of* S. aureus*. However since it is time consuming, expensive, complicated, and difficult to standardize among different laboratories, DNA sequence-based methods have become increasingly popular during the recent years [10, 11]. Genetic analysis of strain types of* S. aureus* can be performed by spa sequence typing. The* spa* gene (approximately 2150 bp) is composed of three regions, namely, the fc protein, the x region, and the c terminal. The spa typing is based on sequencing of the polymorphic x region of protein A and depends on PCR amplification of this hypervariable region. The x region is composed of a variable number of 24 base pair repeats which may differ by spontaneous mutation or deletion and duplication of the repeats. Each repeat is attributed to one alpha-numerical code and the spa type is derived from the order of specific repeats [12–14]. It can be useful in describing the natural population of* S. aureus *strains as well as in outbreak investigations. However sometimes similar or related spa types are located in different clonal lineage which limits the discriminating power of this method [8]. The purpose of this study was to determine the prevalence of antibiotic resistant* S. aureus* isolates and the use of spa, agr, and SCCmec typing to determine the dominant types in Iran.

## 2. Material and Method

Fifty isolates of* S. aureus* were collected from clinical samples of patients who referred to Isfahan's Alzahra hospital (Iran) from January to May 2010. These isolates were obtained from different clinical sources including wound, blood, urine, and sputum.* S. aureus* identification was performed by standard tests including gram staining, catalase, DNase, mannitol fermentation, slide, and tube coagulase. Thereafter they were classified as community-acquired (CA-MRSA) or hospital-acquired (HA-MRSA) based on the patients recorded data.

### 2.1. Susceptibility Test

The susceptibility of* S. aureus *isolates to antimicrobial agents was determined by the disk diffusion method according to the guidelines of the Clinical and Laboratory Standards Institute (CLSI) [15]. The antibiotics utilized were as follows: vancomycin, tetracycline, gentamicin, clindamycin, ciprofloxacin, rifampin, cefoxitin, levofloxacin, and cotrimoxazol.* S. aureus* strain ATCC25923 was used as a control strain for the quality control of antibiotic susceptibility testing.

### 2.2. Molecular Detection of* mecA* Gene

DNA extraction was performed from all isolates using Fermentas DNA kit in accordance with the manufacturer's protocol. PCR was performed for the detection of* mecA* gene using primers displayed in Table 1. PCR conditions were as follows: initial denaturation, 94°C for 5 min, 40 cycles of denaturation at 94°C for 30 s, annealing at 57°C for 45 s, and extension at 72°C for 30 s and final extension at 72°C for 5 min.

### 2.3. Multiplex PCR for SCCmec and Agr Typing

SCCmec typing for 9 isolates resistant to methicillin (MRSA) was determined by multiplex PCR method. Primers shown in Table 1 were used for this purpose. The PCR protocol comprised an initial denaturation step at 94°C for 4 min followed by 30 cycles of denaturation at 94°C for 30 s, annealing step at 55°C for 30 s, and extension at 72°C for 60 s and a final extension step at 72°C for 4 min. Agr typing was also performed on all isolates of* S. aureus* using primers shown in Table 1. PCR conditions were as follows: initial denaturation at 94°C for 5 min followed by 40 cycles of denaturation at 94°C for 40 s, annealing at 60°C for 40 s, and extension at 72°C for 60 s and a final extension at 72°C for 5 min.

### 2.4. Spa Typing

The polymorphic X region of the* spa *gene was amplified from all* S. aureus* isolates using the spa primers exhibited in Table 1. All sequencing reactions were performed at Bioneer (Korea) and then the data were analyzed using MEGA 4 software. Finally, spa types were assigned by Ridom SpaServer (http://spaserver.ridom.de) [12].

## 3. Results

In the present study, from January to May 2010, 50 isolates of* S. aureus* from various clinical specimens including wound (38%), septicemia (26%), UTI (18%), pneumonia (10%), and others (2%) were collected from Alzahra Hospital in Isfahan.

In this study, antimicrobial susceptibility tests were performed by disk diffusion method.* S. aureus* resistance to tetracycline, cotrimoxazol, cefoxitin, clindamycin, ciprofloxacin, gentamicin, levofloxacin, rifampin, and vancomycin was 36%, 22%, 18%, 12%, 12%, 10%, 6%, 6%, and 0%, respectively. In our study, presence of* mecA *gene in all isolates were evaluated by susceptibility test and then confirmed by PCR. Of the 50* S. aureus *isolates, 8 (16%) were MRSA and* mecA *positive. To determine the type of MRSA isolates, SCCmec typing was performed in which 4 (44.4%) were found to be SCCmec type IV, 2 (22.2%) were SCCmec type III, and 2 (22.2%) were SCCmec type I. Agr typing results revealed that 45 (90%) isolates were agr type I, 2 (4%) were agr type III, and 3 (6%) were nontypeable. Ultimately, typing of 50 isolates of* S. aureus* was performed using spa typing method. Genotyping of* spa* gene revealed 22 different spa types with Spa type t7688 (26%) being the most frequent and other types with the following frequencies: t304 (10%), t037 (8%), t005 (8%), t230 (8%), t024 (6%), and t4892 (4%). Spa types including t352, t8015, t937, t138, t436, t439, t045, t2790, t084, t1741, t267, t021, t7685, t5005, and t275 were found only once among the isolates (Table 2).

## 4. Discussion

*S. aureus*, particularly methicillin-resistant* Staphylococcus aureus* (MRSA), is one of the most common causes of infection both in the community and in hospitals. Due to its diverse pathogenicity and high antibiotic resistance, drug therapy has been problematic causing great burdens for patients and healthcare providers. Molecular typing of this bacterium is therefore essential to determine the origin of the strains, its clonal relations, and for epidemiological investigations, playing an important role in the prevention and treatment of infections [16]. In this study, we investigated the antibiotic resistance of* S. aureus *to tetracycline, cefoxitin, clindamycin, ciprofloxacin, gentamycin, cotrimoxazol, levofloxacin, rifampin, and vancomycin using disk diffusion method. Resistance of* S. aureus* to antibiotics was as follows: tetracycline 36%, cefoxitin 18%, clindamycin and ciprofloxacin 12%, gentamicin 10%, cotrimoxazol 22%, levofloxacin and rifampin 6%, and 0% to vancomycin. Resistance rates found in this study were lower than the global average [5, 17, 18].

Several different phenotypic and genotypic methods can be employed for classifying strains used in epidemiological investigations, and for detection and monitoring nosocomial outbreaks. In the current study, we used SCCmec, agr, and spa typing for this purpose [18, 19]. Our results revealed that 16% of the isolates were MRSA and* mecA* positive. MRSA classification requires a thorough understanding of their genetic structure as well as detection of all SCCmec types and carriers of the* mecA* gene. SCCmec typing provides important information about the movable genetic components responsible for resistance to methicillin and it is a marker for differentiation between HA-MRSA and CA-MRSA strains [6, 9, 19]. SCCmec typing was performed on MRSA isolates in which 4 (44.4%) were SCCmec type IV (2 of them were related to t037 spa type and agr types I and III, and the other two isolates were t325 and t005 spa types and agr type I), 2 (22.2%) were SCCmec type III (related to t037 and t138 spa types and agr type I), and 2 (22.2%) were SCCmec type I (related to t005 and t304 spa types and agr type I). The high frequency of SCCmec types IV compared to other SCCmec types may be due to their small size that facilitates their spread among* S. aureus* strains [6]. In our study, 3 isolates with SCCmec type IV belonged to HA-MRSA, whereas types IV and V were shown to belong to CA-MRSA. As expected, in this study we have shown 4 isolates related to SCCmec I and III which belong to HA-MRSA [9]. Similar results were shown by Vindel et al. [17].

In agreement with previous reports from Iran, the majority 45(90%) of the isolates were agr I, 3 (6%) were agr III, and 2 (4%) were nontypeable [4, 5] Similar to many previous studies, the agr type IV was absent in our study [2, 20].

Spa typing is an effective molecular typing technique based on sequencing of only single locus of* S. aureus* and has advantages such as rapidity, ease, and convenience of interpreting the results and exchangeability of results among laboratories and creates a global database based on spa typing for national and international control of* S. aureus.*

The use of spa typing in our study revealed 22 different spa types where Spa type t7688 (26%) was the most frequent followed by Spa type t304. Its global prevalence has been shown to be 0.32% in different countries including Austria, Belgium, Canada, Denmark, Finland, France, Gabon, Germany, Iceland, Sweden, Switzerland, United Arab Emirates, United Kingdom, United States, Lebanon, Netherlands, Norway, South Africa, and Spain (http://spaserver.ridom.de), whereas the frequency of this type was higher (10%) in our study. In addition the frequency of spa t304 has been found to be higher than that reported in previous studies in Iran [21].

Spa types t037, t005, and t230 have been isolated from different parts of the world [1, 8, 21]. However, in our study each one was 8% and Spa types t024 and t4892 were 6% and 4%, respectively. Other spa types including t325, t267, t021, t275, t7685, t045, t005, t439, t138, t937, t436, t8015, t325, t084, t1741, and t5005 were found only once among the isolates. In a study by Wiśniewska et al. the prevalent spa types in Poland were reported to be t003, t151, and t008 [9]. Neela et al. showed that Spa type t037 and SCCmecIII were prevalent in Malaysia [22]. Tokajian et al. found Spa types t044 and t037 and SCCmec IV as the prevalent types in Lebanon [18]. The use of molecular typing in Spain revealed Spa types t067 and t002, agr II, and SCCmec IV to be dominant [17]. In a study conducted by Emaneini et al. Spa type t7685 was shown to be prevalent in Iran [21]. There seems to be a geographical difference in the distribution of various spa types.

## 5. Conclusion

Overall in the current study, spa typing showed 100% type ability. An interesting point to note was the dominance of Spa type t7688 which has been only reported from Iran to date. Moreover the spa types of t084 and t037 are associated with the top ten spa types worldwide between MRSA and MSSA isolates in which t037 is one of the prevalent types in Asian countries. It is our understanding that the current results will aid in the characterization of* S. aureus* in neighboring countries. We suggest the use of additional typing methods such as BURP and MLST to overcome the limitation of a single locus-based molecular typing (spa typing).

## Figures and Tables

**Table 1:** Primers used in this study.

Target	Primer	Sequence	Product size (bp)	Reference
mecA	F	AAAATCGATGGTAAAGGTTGGC	533	[2]
	R	AGTTCTGCAGTACCGGATTTG		

SCCmec	*β*F	ATTGCCTTGATAATAGCCYTCT	937	[3]
	*α*3R	TAAAGGCATCAATGCACAAACACT		
	ccrCF	CGTCTATTACAAGATGTTAAGGATAAT		
	ccrCR	CCTTTATAGACTGGATTATTCAAAATAT	518	
	1272F1	GCCACTCATAACATATGGAA		
	1272R1	CATCCGAGTGAAACCCAAA	1415	
	5RmecA	TATACCAAACCCGACAACTAC		
	5R431	CGGCTACAGTGATAACATCC	359	

Agr	PanF	ATGCACATGGTGCACATGC		[20]
	RI	GTCACAAGTACTATAAGCTGCGAT	439	
	RII	GTATTACTAATTGAAAAGTGCCATAGC	572	
	RIII	CTGTTGAAAAAGTCAACTAAAAGCTC	406	
	RI V	CGATAATGCCGTAATACCCG	659	

Spa	1113F	TAAAGACGATCCTTCGGTGAGC	Variable	[22]
	1514R	CAGCAGTAGTGCCGTTTGCTT		

**Table 2:** Correlation between the different molecular typing methods.

Strain	Sample	*spa* type	*agr* type	*mecA* gene	*Sccmec* type	PVL gene^1^	Antimicrobial resistance^2, 3^
1	Wound	t230	I	−	−	−	−
2	Septicemia	t230	I	−	−	+	−
3	Septicemia	t024	I	−	−	−	−
4	Septicemia	t304	I	−	−	−	tet
5	Urine	t304	I	−	−	−	tet
6	Wound	t4892	I	−	−	+	cot
7	Wound	t024	I	−	−	−	−
8	Wound	t304	I	−	−	−	−
9	CSF	t2790	I	−	−	−	−
10	Wound	t304	I	−	−	+	tet
11	Wound	t024	I	−	−	+	−
12	Wound	t7688	I	−	−	+	tet
13	Blood	t045	I	−	−	+	−
14	Sputum	t005	I	+	IV	+	cx
15	Blood	t7688	I	−	−	−	−
16	Urine	t439	I	−	−	−	−
17	Wound	t7688	I	−	−	−	cx
18	Wound	t005	I	+	I	−	cx
19	Urine	t7688	I	−	−	−	−
20	Wound	t7688	I	−	−	+	−
21	Blood	t7688	I	−	−	−	−
22	Blood	t7688	I	−	−	−	−
23	Wound	t7688	I	−	−	−	−
24	Wound	t7688	I	−	−	+	−
25	Urine	t7688	I	−	−	−	−
26	Wound	t230	I	−	−	−	−
27	Blood	t230	I	−	−	−	−
28	Sputum	t005	I	−	−	−	−
29	Wound	t7688	I	−	−	−	−
30	Wound	t7688	I	−	−	+	−
31	CSF	t7688	I	−	−	−	−
32	Sputum	t4892	I	−	−	−	−
33	Urine	t304	I	+	I	−	cx
34	Wound	t138	I	+	III	+	cx, cot
35	Wound	t037	I	+	III	−	Cip, gen, cd, tet, le, rif, cx, cot
36	Perituen	t037	III	+	IV	−	Cip, gen, cd, tet, le, cx, cot
37	Urine	t937	I	−	−	−	Cip, gen, cd, tet, le
38	Wound	t436	I	−	−	−	gen, cd, tet, cx
39	Wound	t8015	III	−	−	−	gen, cd, tet, cx
40	Abscess	t037	I	+	IV	−	Cip, gen, cd, tet, rif, cx, cot
41	Blood	t352	I	−	−	−	tet, rif, cx
42	Sputum	t084	I	−	−	−	tet, cot
43	Urine	t1741	−	−	−	−	tet
44	Sputum	t005	I	−	−	−	−
45	Wound	t037	I	−	−	−	tet, cot
46	Blood	t267	I	−	−	−	Cip, tet, cot
47	Urine	t021	−	−	−	−	tet
48	Blood	t7685	I	−	−	−	Cip, tet
49	Blood	t5005	I	−	−	−	tet
50	Wound	t275	I	−	−	−	−

^1^PVL: Panton-Valentine Leukocidin.

^2^Oxa: oxacillin, Lev: levofloxacin, Cip: ciprofloxacin, Tet: tetracycline, Cot: cotrimoxazole, Gen: gentamycin, Cli: clindamycin, Rif: rifampicin.

^3^No resistance was observed for vancomycin and minocycline.

## References

[1] Cookson B. D., Robinson D. A., Monk A. B. (2007). Evaluation of molecular typing methods in characterizing a European collection of epidemic methicillin-resistant *Staphylococcus aureus* strains: the HARMONY collection. *Journal of Clinical Microbiology*.

[2] Kilic A., Guclu A. U., Senses Z., Bedir O., Aydogan H., Basustaoglu A. C. (2008). Staphylococcal cassette chromosome *mec*(SCC*mec*) characterization and panton-valentine leukocidin gene occurrence for methicillin-resistant *Staphylococcus aureus* in Turkey, from 2003 to 2006. *Antonie van Leeuwenhoek*.

[3] Koreen L., Ramaswamy S. V., Graviss E. A., Naidich S., Musser J. M., Kreiswirth B. N. (2004). *spa* typing method for discriminating among *Staphylococcus aureus* isolates: implications for use of a single marker to detect genetic micro- and macrovariation. *Journal of Clinical Microbiology*.

[4] Azimian A., Najar-Pirayeh S., Mirab-Samiee S., Naderi M. (2012). Occurrence of methicillin resistant *Staphylococcus aureus* (MRSA) among clinical samples in Tehran-Iran and its correlation with polymorphism of specific accessory gene regulator (*AGR*) groups. *Brazilian Journal of Microbiology*.

[5] Peerayeh S. N., Azimian A., Nejad Q. B., Kashi M. (2009). Prevalence of *agr* specificity groups among *Staphylococcus aureus* isolates from university hospitals in Tehran. *Laboratory Medicine*.

[6] Deurenberg R. H., Stobberingh E. E. (2008). The evolution of *Staphylococcus aureus*. *Infection, Genetics and Evolution*.

[7] Wong H., Louie L., Lo R. Y. C., Simor A. E. (2010). Characterization of *Staphylococcus aureus* isolates with a partial or complete absence of *Staphylococcal* cassette chromosome elements. *Journal of Clinical Microbiology*.

[8] Strommenger B., Braulke C., Heuck D. (2008). *spa* typing of *Staphylococcus aureus* as a frontline tool in epidemiological typing. *Journal of Clinical Microbiology*.

[9] Wiśniewska K., Szewczyk A., Piechowicz L., Bronk M., Samet A., Świeć K. (2012). The use of *spa* and phage typing for characterization of clinical isolates of methicillin-resistant *Staphylococcus aureus* in the University Clinical Center in Gdańsk, Poland. *Folia Microbiologica*.

[10] Arakere G., Nadig S., Swedberg G., Macaden R., Amarnath S. K., Raghunath D. (2005). Genotyping of methicillin-resistant *Staphylococcus aureus* strains from two hospitals in Bangalore, South India. *Journal of Clinical Microbiology*.

[11] Moodley A., Stegger M., Bagcigil A. F. (2006). *spa* typing of methicillin-resistant *Staphylococcus aureus* isolated from domestic animals and veterinary staff in the UK and Ireland. *Journal of Antimicrobial Chemotherapy*.

[12] Harmsen D., Claus H., Witte W., Rothgänger J., Turnwald D., Vogel U. (2003). Typing of methicillin-resistant *Staphylococcus aureus* in a university hospital setting by using novel software for *spa* repeat determination and database management. *Journal of Clinical Microbiology*.

[13] Mitani N., Ohnishi M., Masutani T., Murakawa K., Okamoto Y. (2002). Molecular typing of methicillin-resistant *Staphylococcus aureus* by protein A gene sequencing. *Japanese Journal of Infectious Diseases*.

[14] Shopsin B., Gomez M., Montgomery S. O. (1999). Evaluation of protein A gene polymorphic region DNA sequencing for typing of *Staphylococcus aureus* strains. *Journal of Clinical Microbiology*.

[15] Boye K., Bartels M. D., Andersen I. S., Møller J. A., Westh H. (2007). A new multiplex PCR for easy screening of methicillin-resistant *Staphylococcus aureus* SCC*mec* types I-V. *Clinical Microbiology and Infection*.

[16] Wildemauwe C., de Brouwer D., Godard C. (2010). The use of spa and phage typing for characterization of a MRSA population in a Belgian hospital: comparison between 2002 and 2007. *Pathologie Biologie*.

[17] Vindel A., Cuevas O., Cercenado E. (2009). Methicillin-resistant *Staphylococcus aureus* in Spain: molecular epidemiology and utility of different typing methods. *Journal of Clinical Microbiology*.

[18] Tokajian S. T., Khalil P. A., Jabbour D. (2010). Molecular characterization of *Staphylococcus aureus* in Lebanon. *Epidemiology & Infection*.

[19] Vainio A., Kardén-Lilja M., Ibrahem S. (2008). Clonality of epidemic methicillin-resistant *Staphylococcus aureus* strains in Finland as defined by several molecular methods. *European Journal of Clinical Microbiology & Infectious Diseases*.

[20] Ghaznavi-Rad E., Shamsudin M. N., Sekawi Z. (2010). Predominance and emergence of clones of hospital-acquired methicillin-resistant *Staphylococcus aureus* in Malaysia. *Journal of Clinical Microbiology*.

[21] Emaneini M., Khoramrooz S. S., Taherikalani M., Jabalameli F., Aligholi M. (2011). Molecular characterization of *Staphylococcus aureus* isolated from children with adenoid hypertrophy: emergence of new *spa* types t7685 and t7692. *International Journal of Pediatric Otorhinolaryngology*.

[22] Neela V., Moghaddam H. G., van Belkum A., Horst-Kreft D., Mariana N. S., Rad E. G. (2010). First report on methicillin-resistant *Staphylococcus aureus* of *spa* type T037, sequence type 239, SCC*mec* type III/IIIA in Malaysia. *European Journal of Clinical Microbiology & Infectious Diseases*.

